# Explainable Mortality Prediction for Liver Transplant Candidates with Hepatocellular Carcinoma: A Supervised Clustering Approach

**DOI:** 10.34133/hds.0295

**Published:** 2026-01-13

**Authors:** Abdelghani Halimi, Nesma Houmani, Sonia Garcia-Salicetti, Ilias Kounis, Audrey Coilly

**Affiliations:** ^1^ SAMOVAR, Télécom SudParis, Institut Polytechnique de Paris, 91120 Palaiseau, France.; ^2^ Chaire BOPA, Rue de la Chapelle de l’Hôpital, Villejuif, France.; ^3^ AP-HP, Hôpital Paul Brousse, Centre Hépato-Biliaire, Inserm UMR-S 1193, Université Paris-Saclay, Villejuif, France.

## Abstract

**Background:** Accurate mortality prediction for liver transplant candidates with hepatocellular carcinoma (HCC) remains a critical challenge. Traditional scoring systems, including Child–Pugh, Albumin–Bilirubin, Model for End-Stage Liver Disease (MELD), MELD-Na, MELD 3.0, and Alpha-fetoprotein scores, are widely used but often fail to provide precise risk assessments. This limitation arises from the dual burden of liver dysfunction and tumor progression, which complicates prognosis. Consequently, there is a need for a comprehensive approach addressing both considerations to better manage HCC patients. **Methods:** We propose an advanced machine learning-based scoring system exploiting Ensemble Learning and SHapley Additive exPlanations (SHAP) for a better understanding of key mortality risk factors. SHAP offers valuable insights into the decision-making process by providing both global and local explanations. By embedding SHAP values in the Uniform Manifold Approximation and Projection space, we perform supervised clustering to infer latent subgroups, providing a higher granularity on the contribution of key variables for mortality risk assessment. **Results:** Our system based on LightGBM outperforms conventional scores leveraging only 8 relevant variables selected by SHAP analysis. These variables respond to the challenging dual risk problem set in this work. With supervised clustering, we uncover 7 subgroups showing an increasing mortality risk level and a fine assessment of risk factors’ contribution. **Conclusion:** By contrast to existing studies, our approach offers an integrative data-driven framework for handling the dual risk challenge set by HCC patients with liver dysfunction. Also, it provides a valuable tool for a more precise risk evaluation that may guide treatment decisions and help monitoring patient progression.

## Introduction

Liver cancer is a global public health concern, ranking as the third leading cause of cancer-related deaths worldwide in 2020 [1]. Hepatocellular carcinoma (HCC), the most prevalent type of liver cancer, often requires liver transplantation (LT) as the definitive therapy for early-stage disease. Nevertheless, the scarcity of donor organs underscores the critical need for efficient prioritization strategies to manage transplant waitlists effectively.

The current liver allocation system is designed to prioritize LT candidates based on their estimated risk of waitlist mortality. This system relies on statistically derived scores that integrate key laboratory parameters, such as serum sodium, bilirubin, creatinine, and albumin. However, the accurate prediction of waitlist mortality in patients with HCC is particularly challenging due to the dual risks they face: death from liver failure and dropout from the waitlist due to tumor progression. Traditional scores like Child–Pugh [2], Albumin–Bilirubin (ALBI) [3], Model for End-Stage Liver Disease (MELD) [4], MELD-Na [5], and MELD 3.0 [6] effectively assess the severity of liver disease and mortality risk from cirrhosis but do not account for tumor-related risks. Conversely, Alpha-fetoprotein (AFP) score [7] focuses on tumor progression but overlooks the competing risk of liver failure. This creates an unmet clinical need for an integrated approach that evaluates both risks simultaneously. In this regard, other statistically derived scores, namely, hazard associated with LT for HCC (HALT-HCC) [8] and the Mehta model [9], were proposed to take into account both types of risks.

However, all these scores rely on linear models assigning fixed weights to variables, regardless of their interactions with other factors. Also, they exclusively include variables measured at a single time point, not taking into account the progression of the disease. Both facts limit accuracy and personalization of risk assessment. These limitations motivate the exploration of more flexible approaches for LT risk stratification, in line with recent work on integrating machine learning (ML) with statistical prediction methods [10].

ML has emerged as a promising solution to predict waitlist mortality, by leveraging high-dimensional and multimodal data to capture intricate patterns that traditional scores may miss [11–13]. Several studies demonstrated the effectiveness of ML in predicting waitlist mortality for non-HCC patients comparatively to conventional statistically derived models such as MELD-based scores [14–22].

Conversely, only one study has specifically addressed waitlist mortality prediction in HCC patients [20]. The authors proposed 2 models based on optimized decision trees, one for non-HCC candidates and the other for HCC candidates. The results showed improved mortality prediction over MELD-Na (area under the curve 0.859 vs. 0.841). Nevertheless, these results are based on a mixed HCC and non-HCC cohort.

The above-mentioned studies on ML suffer from several limitations: (a) some incorporated numerous features without prior relevance assessment, risking reliance on highly correlated features that may obscure the identification of key patterns and reduce interpretability [15–17,19–21]; (b) no study examined the contribution of features to the model’s predictions, thereby limiting their applicability in clinical settings; and (c) all focused on assessing waitlist mortality risk at the population level, limiting their potential for personalized insight, which is crucial for advancing precision medicine.

This study addresses these limits by proposing an advanced ML framework with 2-fold purposes. First, we introduce a new scoring system to predict 3-month waitlist mortality in HCC patients, based on ensemble learning (EL). We couple our model with the SHapley Additive exPlanations (SHAP) method to identify critical risk factors and investigate their contribution to risk prediction. Our approach aims not only at improving predictive accuracy, but also at achieving a more profound clinical interpretability of patients’ risk through SHAP analysis. Second, for a more nuanced understanding of the underlying risk factor interdependencies, we adopt a supervised clustering approach by performing *k*-medoids clustering in an embedded SHAP values space using the Uniform Manifold Approximation and Projection (UMAP) method. This approach allows uncovering distinct and clinically meaningful subgroups of patients with specific profiles.

To the best of our knowledge, this work represents the first comprehensive study on ML models for an in-depth analysis of waitlist mortality among HCC transplant candidates.

## Methods

### Study population

This study exploits data from the Organ Procurement and Transplantation Network (OPTN) and the United Network for Organ Sharing (UNOS), contained in the Standard Transplant Analysis and Research (STAR) file. We considered several observations (visits) from adult patients registered from 2002 February 27 to 2023 September 30 and not listed for multiorgan transplants in order to mitigate potential confounding factors.

The study population consists of adult patients with HCC exception status who either (a) remained on the waitlist beyond 3 months, referred to as the “on waiting list” group; or (b) died while on the waitlist or were removed for being too sick for transplantation within the 3-month period, referred to as “waitlist mortality”.

We excluded patients who received the transplant before the 3-month period as in Refs. [14,19,20], because it was not possible to ascertain their true outcome in the absence of transplantation.

The cohort includes clinical, laboratory, and disease-related variables from 11,647 patients: 11,199 patients “on waiting list” and 448 patients of the “waitlist mortality” group. Variable descriptions and cohort demographics are given in Tables S1 and S2, respectively.

### Data preprocessing and feature engineering

The study focuses on variables recorded prior to either transplant or removal from the waiting list (see Table S1). To capture the dynamic nature of patients’ health, we computed the difference (DIFF) between consecutive measurements for each patient across 6 key laboratory variables involved in traditional scores: serum sodium, creatinine, albumin, bilirubin, AFP, and International Normalized Ratio (INR). This results in dynamic variables tracking the direction of intrapatient change (positive for increases, negative for decreases). Given the nonuniform timing of measurements in the UNOS STAR file, these were interpreted as markers of disease trajectory rather than exact rates of change per unit time. Finally, the largest tumor size was determined by selecting the largest value among the 5 tumor sizes recorded in the OPTN database.

For numerical variables, missing values (less than 7% of the data) were imputed with the mean values of each respective class. For categorical variables, we removed observations with missing data, since they represented less than 0.1% of the total data. One-hot encoding [23], with the first category omitted to prevent multicollinearity, was used to transform categorical variables for model training.

### Experimental design

**Fig. 1.**
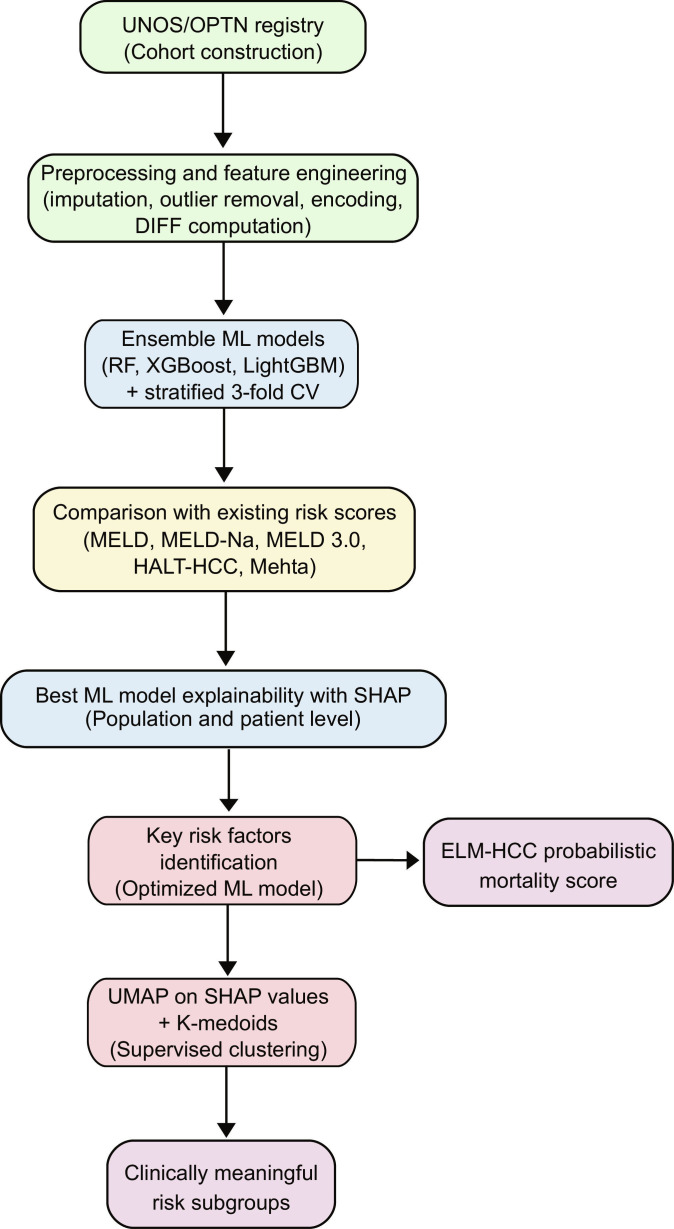
Proposed workflow for HCC mortality risk assessment and stratification.

This study targets predicting mortality on the waiting list using EL tree-based models, which are particularly effective for handling tabular and heterogeneous data [14,24,25]. EL paradigm relies on the combination of multiple weak learners, thereby improving overall performance. Figure 1 presents the entire pipeline of the proposed scoring system.

For comparative purposes, we used 3 tree-based ensemble models: Random forest (RF) [26], XGBoost [27], and LightGBM [28]. We evaluated them in 2 settings: first, using only the original static variables (25 features), and then incorporating dynamic (DIFF) variables (31 features). We also compared the performance of EL-based models with those of the traditional statistically derived scores.

### Data balancing and cross-validation

Given the unbalanced nature of the dataset, we down-sampled the majority class (“on waiting list”) into 30 partitions with equivalent size to the minority class (“mortality waitlist”). Each partition contains different patients from the majority class. For each balanced subset, we performed a 3-fold cross-validation protocol, ensuring that all observations from a single patient were either in the training set or the test set.

### Hyperparameter tuning

The optimal configuration for the EL-based classifiers was determined using a grid search for each hyperparameter described in Table 1.

**Table 1.** Hyperparameter space

Methods	Hyperparameters	Grid
Random forest	Number of trees	50 to 250 (step of 10)
	Max tree depth	[None, 2, 3, 4]
XGBoost	Number of estimators	5 to 50 (step of 5)
	Max depth	2 to 16 (step of 2)
	Learning rate	[0.2, 0.3, 0.4]
LightGBM	Number of estimators	10 to 200 (step of 10)
	Max depth	2 to 16 (step of 2)

### Evaluation metrics

Models’ performance was assessed with several metrics, including area under the receiver operating curve (AUROC), sensitivity (percentage of patients from “waitlist mortality” correctly classified), and specificity (percentage of patients “on waiting list” correctly classified). These metrics were computed for each of the 3 folds for the 30 subsets and then averaged across all of them. The optimal decision threshold was that maximizing both sensitivity and specificity.

### Feature selection and score construction

After evaluating the classifiers using the whole set of features, we assessed their performance using only the most pertinent features, selected with 2 methods: Gain importance and SHAP global importance [29–32]. The resulting optimal model was used to provide a novel probabilistic mortality score for HCC patients based on EL modeling, referred to as ELM-HCC.

### Explainability with SHAP

SHAP [33,34] was additionally used to explain the model’s predictions. Indeed, the computed SHAP values quantify the contribution of each feature to the classifier’s prediction, by comparing predictions with and without the feature across all possible feature combinations. This allows identifying the most relevant features while also providing insights into their impact on the predicted outcomes.

### Supervised clustering analysis

Finally, moving from a population level prediction to a subgroup-specific analysis, we performed a *K*-medoids [35] clustering on UMAP embeddings of SHAP values. This methodology is conceptually aligned with “supervised clustering” [36], since SHAP values, rather than raw data, are clustered. By weighting features based on their importance, SHAP minimizes irrelevant variations and standardizes feature data, improving interpretability and cluster differentiation [36–38]. The reduction in dimensionality with UMAP enables more effective clustering [39–41] and helps for cluster visualization. For UMAP implementation, we set the number of nearest neighbors to 100 and the minimum distance to 0.05 to balance local and global structures in the low-dimensional representation. The choice of the number of clusters was first guided by quantitative indices (Silhouette coefficient [42] and Davies–Bouldin index [43]) and then confirmed through SHAP-based inspection of cluster characteristics, ensuring clinically interpretable risk profiles.

### Traditional mortality risk scores

We analyzed 8 existing scores that incorporate liver function, tumor burden, and overall patient condition. Each score was computed per observation as detailed in the sequel.

#### ALBI score

It is used for assessing liver dysfunction in patients with chronic liver disease and HCC [3]. ALBI score involves 2 measured liver function parameters, bilirubin and albumin, as follows:

$$\text{ALBI}=\left(0.66\times {\text{log}}_{10}\left(\text{Bilirubin}\right)\right)-\left(0.085\times \text{Albumin}\right)$$ (1)

#### Child–Pugh score

It is used to assess liver function and estimate life expectancy [2]. It is calculated as a sum of points assigned to 5 variables, namely, bilirubin, albumin, INR, ascites, and encephalopathy. Each variable is valued 1, 2, or 3 points based on its severity.

#### MELD scores

MELD score and its variants [4–6] are widely used to predict mortality risk in patients with end-stage liver disease. They are computed as follows:

$$\begin{array}{c} \begin{array}{ll} \text{MELD}\;= & \;3.78\times {\text{log}}_{10}\left(\text{Bilirubin}\right)+11.2\times {\text{log}}_{10}\left(\mathrm{INR}\right)\\ & \;+\;9.57\times {\text{log}}_{10}\left(\text{Creatinine}\right)+6.43 \end{array} \end{array}$$ (2)

$$\begin{array}{c} \begin{array}{ll} \text{MELD}-\mathrm{Na}\;= & \;\text{MELD}+1.32\times \left(137-\text{Sodium}\right)\\ & \;-\left[0.033\times \text{MELD}\times \left(137-\text{Sodium}\right)\right] \end{array} \end{array}$$ (3)

$$\begin{array}{c} \begin{array}{ll} \text{MELD}3.0 & =1.33\times \left(\text{Female}\right)+4.56\times {\text{log}}_{10}\left(\text{Bilirubin}\right)\\ & -\;0.24\times \left(137-\text{Sodium}\right)\times {\text{log}}_{10}\left(\text{Bilirubin}\right)\\ & +\;9.09\times {\text{log}}_{10}\left(\mathrm{INR}\right)+0.82\times \left(137-\text{Sodium}\right)\\ & +\;11.14\times {\text{log}}_{10}\left(\text{Creatinine}\right)\\ & +\;1.85\times \left(3.5-\text{Albumin}\right)\\ & -\;1.83\times \left(3.5-\text{Albumin}\right)\times {\text{log}}_{10}\left(\text{Creatinine}\right)\\ & +\;6 \end{array} \end{array}$$ (4)

#### AFP score

It is used to predict HCC-related mortality and recurrence risk [7]. The total score is determined by summing the points assigned to the 3 tumor-related variables, namely, largest tumor diameter, number of tumor nodules, and AFP level.

#### Hazard associated with LT for HCC

It was developed to predict mortality risk in HCC patients, integrating tumor burden, AFP levels, and MELD-Na [8].

$$\begin{array}{c} \begin{array}{ll} \text{HALT}-\mathrm{HCC}\;= & 1.27\times \sqrt{\text{Tumor}\;{\text{Number}}^{2}+\text{Tumor}\;{\text{Size}}^{2}}\\ & \;+1.85\times {\text{log}}_{10}\left(\mathrm{AFP}\right)\\ & \;+0.26\times \text{MELD}‐\mathrm{Na} \end{array} \end{array}$$ (5)

#### Mehta model

It is a composite score designed to refine transplant prioritization [9]. It is computed as follows:

$$\begin{array}{c} \begin{array}{c} \begin{array}{cc} \text{Mehta Model}=\left(\text{MELD}‐\text{Na}-10\right)+3\times \left(\text{Child}\;–\;\text{Pugh Score}-5\right)\\ & \;+\text{AFP Points}+\text{Tumor Points} \end{array} \end{array} \end{array}$$ (6)

AFP and tumor points are assigned based on severity, with higher values correlating with increased mortality risk.

### Statistical analysis

To evaluate whether any significant differences exist between groups, we first applied the Kruskal–Wallis test (α=0.05) [44,45]. If significant differences are found, we then conducted pairwise Dunn’s tests with the Benjamini–Hochberg stepwise adjustment (α=0.05) to identify which specific subgroups differ significantly, while controlling for the false discovery rate [46].

## Results

### Risk score performance

Table 2 reports the predictive performance of traditional and EL-based risk scores for waitlist mortality in HCC patients.

**Table 2.** Performance metrics for traditional scores and the 3 EL models with different variable counts. The table cells contain mean values and 95% confidence intervals. Best performance values are displayed in bold.

	AUROC	Accuracy (%)	Sensitivity (%)	Specificity (%)	Precision (%)	F1-score (%)
AFP score	0.592	58.67	35.61	**81.99**	67.70	45.79
	[0.585, 0.598]	[58.02, 59.31]	[33.94, 37.29]	[80.54, 83.45]	[66.14, 69.27]	[44.31, 47.27]
Child–Pugh Score	0.705	66.79	56.65	76.93	72.31	62.64
	[0.698, 0.712]	[66.24, 67.34]	[54.52, 58.77]	[74.86, 78.99]	[70.89, 73.72]	[61.51, 63.76]
ALBI score	0.707	67.08	59.31	74.64	70.90	64.13
	[0.700, 0.714]	[66.49, 67.67]	[57.72, 60.91]	[72.89, 76.40]	[69.71, 72.08]	[63.18, 65.08]
MELD score	0.734	69.63	62.24	76.94	73.82	66.98
	[0.727, 0.741]	[69.09, 70.17]	[60.52, 63.96]	[75.20, 78.69]	[72.52, 75.13]	[66.01, 67.95]
MELD-Na score	0.743	71.05	63.88	78.39	75.28	68.74
	[0.736, 0.750]	[70.46, 71.64]	[62.64, 65.12]	[76.95, 79.82]	[73.91, 76.64]	[67.94, 69.55]
MELD 3.0 score	0.750	71.06	64.90	77.30	74.70	69.08
	[0.743, 0.757]	[70.44, 71.67]	[63.63, 66.17]	[75.77, 78.83]	[73.36, 76.04]	[68.27, 69.90]
HALT-HCC	0.763	70.80	72.17	69.30	70.93	71.05
	[0.757, 0.768]	[70.31, 71.29]	[70.52, 73.82]	[67.48, 71.11]	[69.85, 72.02]	[70.29, 71.81]
Mehta model	0.782	72.97	71.78	74.21	73.95	72.53
	[0.775, 0.788]	[72.37, 73.57]	[70.38, 73.18]	[72.86, 75.56]	[72.83, 75.08]	[71.68, 73.38]
RF (25 variables)	0.796	73.79	72.41	75.24	74.97	73.32
	[0.789, 0.802]	[73.19, 74.40]	[71.01, 73.80]	[73.80, 76.68]	[73.80, 76.14]	[72.51, 74.14]
XGBoost (25 variables)	0.790	73.63	71.78	75.34	74.94	73.03
	[0.783, 0.797]	[73.01, 74.26]	[70.40, 73.15]	[73.96, 76.72]	[73.87, 76.01]	[72.20, 73.87]
LightGBM (25 variables)	0.790	73.56	72.16	74.99	74.78	73.10
	[0.782, 0.797]	[72.96, 74.15]	[70.80, 73.52]	[73.47, 76.51]	[73.59, 75.96]	[72.34, 73.87]
RF (31 variables)	0.823	75.80	75.70	75.83	76.25	75.63
	[0.817, 0.830]	[75.21, 76.39]	[74.15, 77.25]	[74.45, 77.21]	[75.20, 77.30]	[74.79, 76.47]
XGBoost (31 variables)	0.825	75.72	77.22	74.23	75.37	76.03
	[0.819, 0.832]	[75.19, 76.24]	[76.02, 78.43]	[72.93, 75.53]	[74.39, 76.36]	[75.39, 76.68]
LightGBM (31 variables)	0.826	75.48	**77.42**	73.42	75.00	75.89
	[0.820, 0.831]	[74.96, 76.00]	[76.13, 78.72]	[71.89, 74.95]	[73.96, 76.05]	[75.26, 76.53]
ELM-HCC (8 variables)	**0.835**	**76.32**	77.14	75.64	**76.38**	**76.45**
	[0.829, 0.840]	[75.81, 76.82]	[75.85, 78.44]	[74.29, 76.99]	[75.32, 77.44]	[75.81, 77.09]

Results indicate that while the ALBI score shows better performance than the Child–Pugh and AFP scores, the MELD-based scores exhibit superior performance. Notably, we observe that performance improves with the evolution of the MELD-based scores, with MELD 3.0 outperforming both the MELD and MELD-Na. Nevertheless, all these scores present an imbalance between sensitivity and specificity.

The HALT-HCC and Mehta models outperform the previous scores, with the Mehta model achieving the highest AUROC of 0.782, followed by HALT-HCC at 0.763. Importantly, both models demonstrate a more balanced trade-off between sensitivity and specificity.

When training RF, XGBoost, and LightGBM on the 25 variables, accuracy surpasses that of traditional scores. RF achieves the highest AUROC of 0.796, with a balance between sensitivity (72.41%) and specificity (75.24%).

Furthermore, on the 31 variables including DIFF variables, EL models deliver even better performance. Among them, LightGBM achieves the highest AUROC (0.826) and sensitivity (77.42%), making it the most effective model for identifying high-risk patients.

### Identification of key risk factors

**Fig. 2.**
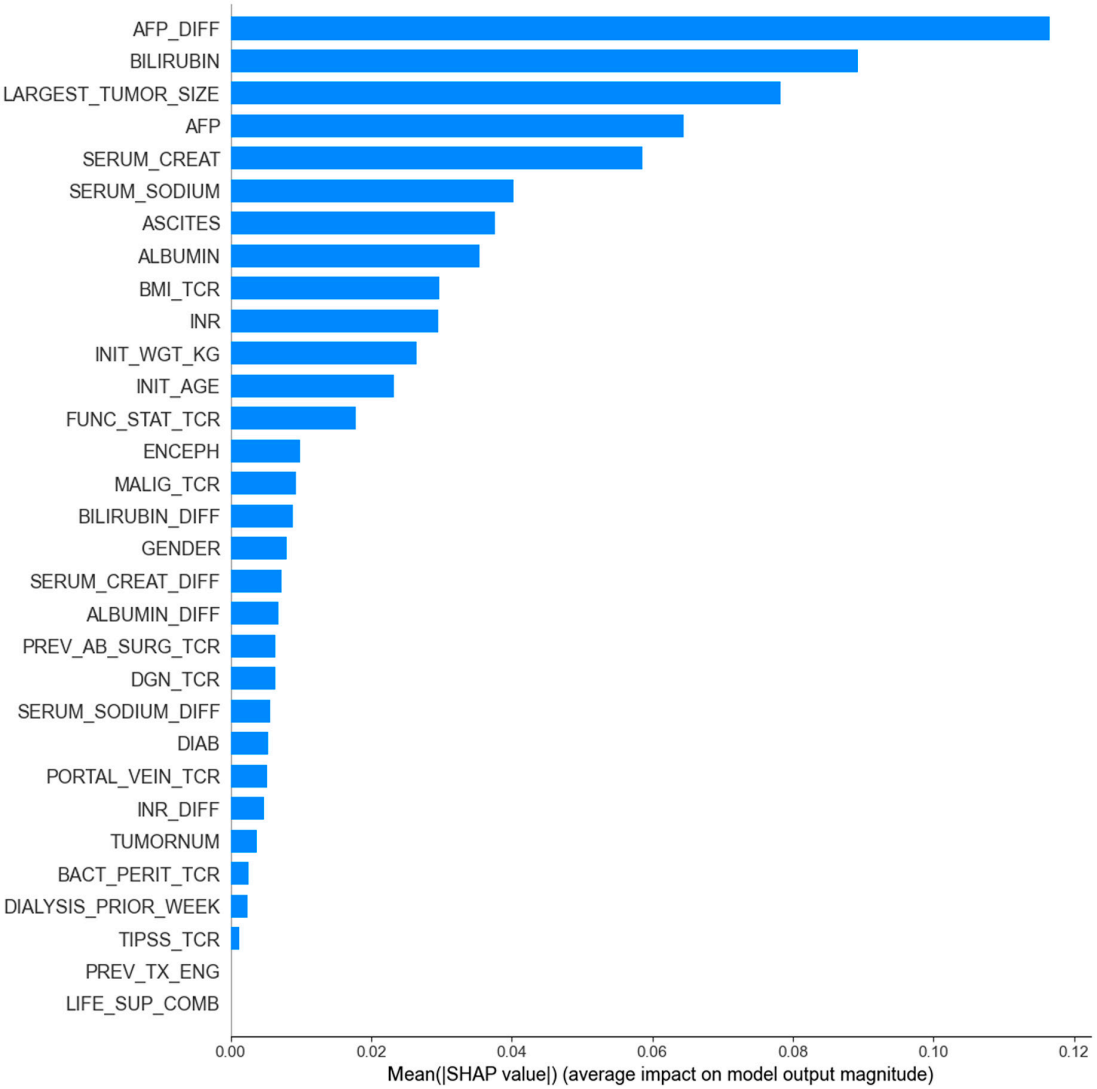
Feature importance of the LightGBM model obtained using SHAP global importance.

Since LightGBM outperformed the other models, we applied feature selection only to this model using Gain importance and SHAP methods. Figure 2 displays the 31 features according to their importance when using SHAP.

Overall, the top 10 variables are identified as the most important predictors by both Gain importance and SHAP global importance methods. Interestingly, key variables incorporated in the MELD-based scores, including bilirubin, creatinine, serum sodium, albumin, and INR, emerge as critical risk factors across both methods. Furthermore, variables related to tumor burden and liver function, namely, largest tumor size, AFP levels, and ascites, also rank among the top predictors of mortality with both methods.

We assessed the performance of LightGBM against the number of features, following the feature ranking previously obtained with Gain importance and SHAP. Based on the ranking given by SHAP, the model’s performance reached an AUROC of 0.835 with the top 8 features, then began to decline. In contrast, the Gain importance ranking showed an AUROC of 0.812 with 8 variables, reaching a maximum of 0.828 when up to 12 variables were included. For this reason, we have chosen to consider the ranking given by SHAP, with 8 features as the optimal cutoff point. The optimal LightGBM trained on such 8 variables generates our ELM-HCC score; its performance is given in Table 2.

Interestingly, this reduced set of variables achieves a better AUROC than the full set of 31 variables, with an AUROC of 0.835 (against 0.826). This result highlights the strong predictive power of the selected 8 variables.

It is noteworthy that AFP_DIFF was found among key relevant features, which highlights the importance of incorporating dynamic information. Moreover, the selected 8 variables, except AFP_DIFF, are all considered by the statistically derived scores. However, these scores did not exploit them concurrently within a unified predictive framework: indeed, each score integrates a subset of these variables, limiting their ability to fully capture the complex interaction between key risk factors and their combined impact on patient outcomes.

### Explanation of LightGBM predictions using SHAP

**Fig. 3.**
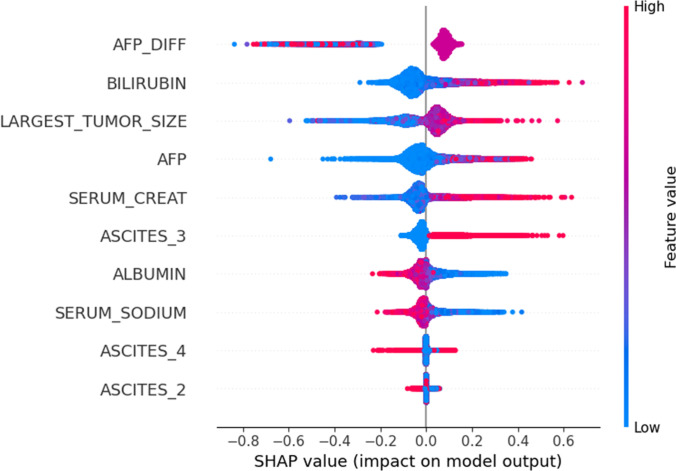
SHAP summary plot of the LightGBM model.

The SHAP summary plot in Fig. 3 shows how each feature (among the 8 selected) contributes to the ELM-HCC score predictions on the whole population.

For the categorical variable ascites, the plot shows the contribution of its distinct categories (see Table S2), with the first category omitted due to one-hot encoding, as noted in Methods. In Fig. 3, the *Y*-axis displays the feature names in order of importance from top to bottom and the *X*-axis displays the SHAP value representing the impact of the feature on the model’s outcome. A positive SHAP value implies an increase in mortality risk, and conversely, a negative SHAP value indicates a decrease in mortality risk. The color is an indicator of each feature’s range, where red indicates high features values and blue indicates low values.

The SHAP summary plot shows that mid-range AFP_DIFF values (in purple), corresponding to stagnant AFP levels, are associated with positive SHAP values, indicating increased mortality risk. Additionally, higher values of bilirubin, largest tumor size, AFP, creatinine, and ascites_3 presence (moderate degree of ascites) are associated with positive SHAP values. This indicates that higher values of such features imply a higher probability of mortality on the waiting list. Conversely, high values of albumin and serum sodium are associated with negative SHAP values, suggesting that elevated levels of these variables correspond to a reduced risk of mortality. These findings highlight that all variables do not contribute to the mortality risk prediction in the same way, emphasizing the complex and variable-specific influence on predictions.

### Risk stratification and subgroup analysis

To uncover the complex relationships among variables and identify latent subgroups that may not be immediately apparent, our approach leverages the SHAP values derived from the LightGBM model trained on the relevant 8 features. We computed SHAP values for the 30 balanced subsets, then selected the subset with the highest AUROC since it conveys the most accurate feature importance evaluation. The corresponding SHAP values were then embedded into a 3-dimensional UMAP space.

**Fig. 4.**
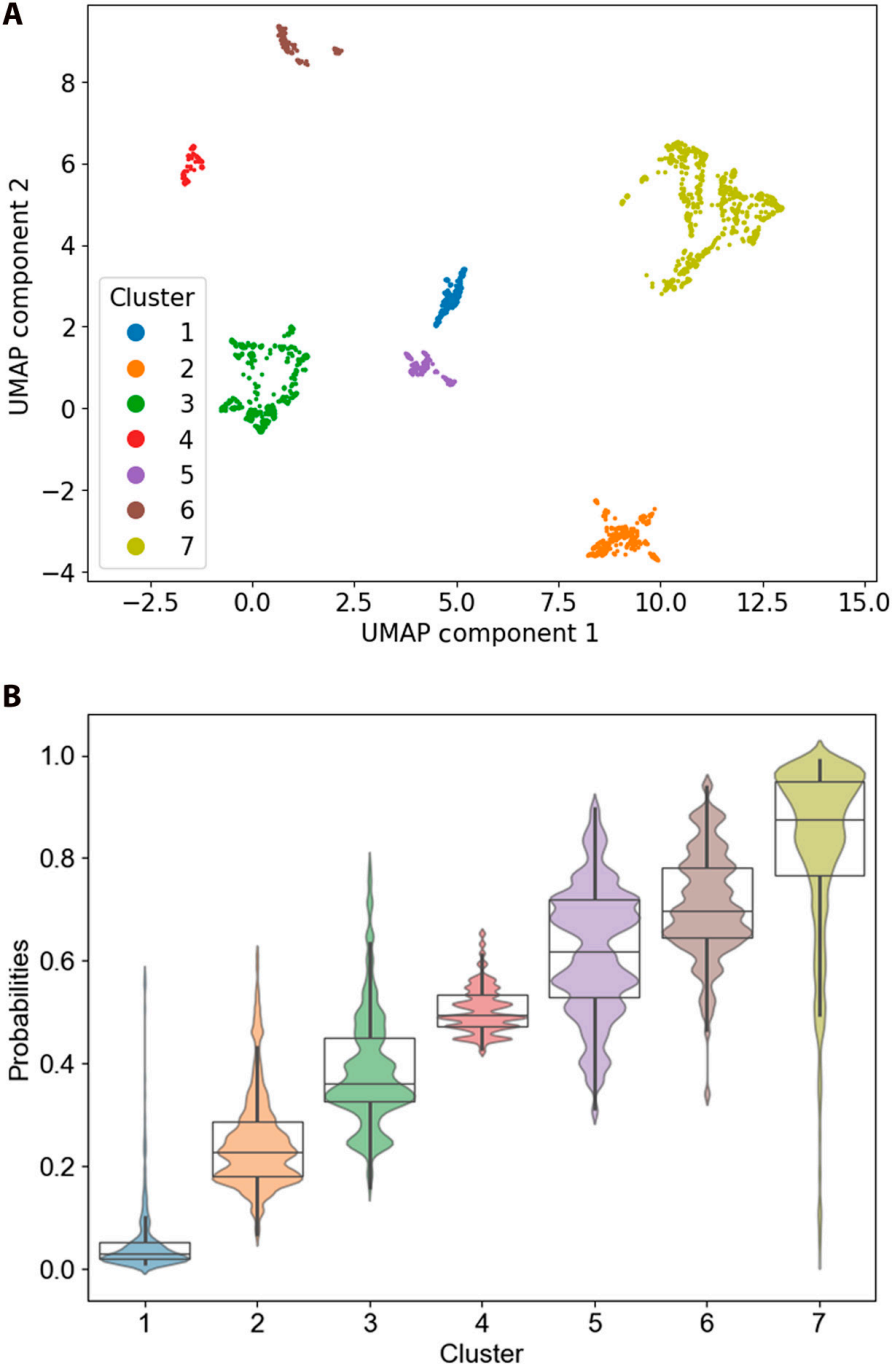
(A) UMAP 2D visualization of the clusters found by *K*-medoids on the SHAP-embedded values, and (B) boxplots and swarm plots of mortality probabilities of the observations across the 7 clusters.

Patients’ observations (visits) are then clustered into distinct subgroups by applying *K*-medoids algorithm within the 3-dimensional UMAP space. To determine the optimal number of clusters, we used 2 cluster validity indices, the Silhouette coefficient [42] and the Davies–Bouldin index [43]. Both indices indicated an optimal partitioning of the data into 7 clusters. Figure 4A shows the 7 clusters visualized in a 2-dimensional UMAP space, and Fig. 4B displays the distribution of the probabilistic ELM-HCC score across these clusters.

Figure 4B clearly shows a mortality risk stratification with a progressive increase in mortality probability from Cluster 1 to Cluster 7. The statistical analysis showed significant differences between clusters (Kruskal–Wallis test, *P* < 0.05). Dunn’s test revealed significant differences in mortality probabilities between all cluster pairs (*P* < 0.05). These findings underscore a distinct risk profile in each cluster.

**Fig. 5.**
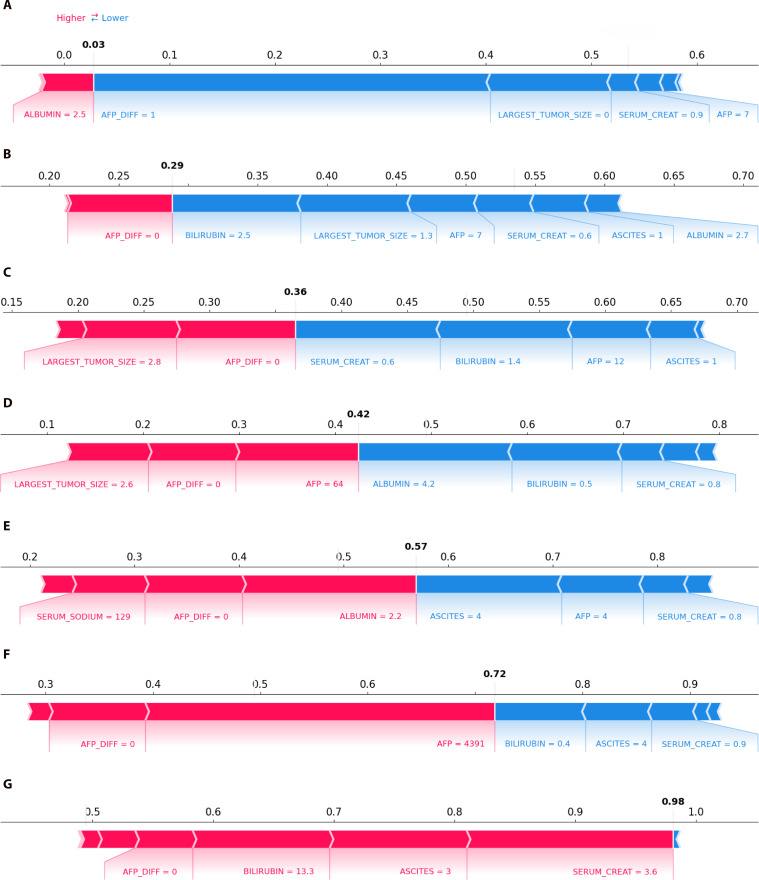
SHAP force plots for the 7 medoids, each representing one cluster: (A) Cluster 1, (B) Cluster 2, (C) Cluster 3, (D) Cluster 4, (E) Cluster 5, (F) Cluster 6, and (G) Cluster 7. Mortality probability for each medoid is in bold. Features in red increase risk (white arrows pointing right), while features in blue decrease risk (white arrows pointing left).

Further analysis based on the Kruskal–Wallis test revealed differences in variables across clusters. For a better insight into the specific variables that contribute to the characterization of clusters, we display in Fig. 5 the SHAP force plots of the 7 medoids representing the 7 clusters previously identified. Each medoid corresponds to a particular observation (visit) that is the closest on average to all the other observations belonging to the same cluster. In each force plot, we represent the mortality probability in bold black font, and underneath the values of each variable at the corresponding visit. The features that contribute to increase the mortality probability are represented in red (positive Shapley values), while those in blue refer to those contributing to decrease the mortality probability (negative Shapley values).

The ELM-HCC score associated to the medoids progressively increase from Cluster 1 to Cluster 7 (Fig. 5A to G), with probabilities of 0.03, 0.29, 0.36, 0.42, 0.57, 0.72, and 0.98, respectively. This trend is consistent with the ranking observed in the boxplots (Fig. 4B), reinforcing the validity of our clustering approach.

The red arrows in the plots signify factors associated with an increased risk of mortality, while the blue arrows denote factors linked to a decreased risk. Overall, high levels of serum creatinine, largest tumor size, bilirubin, and AFP are strongly associated with increased mortality risk, while lower values of such variables correspond to a decreased risk. The force plots reflect this shift, as the color for these variables changes from blue (lower risk) to red (higher risk) as their values increase, aligning with the global trend seen in Fig. 3.

Notably, Cluster 7, representing the highest-risk group, exhibits the highest bilirubin and creatinine, as well as moderate degree of ascites (Ascites_3), indicating severe liver dysfunction. Conversely, Clusters 1 and 2, which correspond to the lowest-risk groups, are primarily distinguished by the smallest tumor size and AFP levels, along with a better preserved liver function, reflected by low bilirubin, low creatinine, and absence of ascites (Ascites_1). Clusters 3 and 4 exhibit profiles similar to Clusters 1 and 2, but differ in having larger tumor size and higher AFP values. Cluster 5 presents low albumin and serum sodium, accompanied by low AFP, suggesting a transitional stage of liver failure. Cluster 6 differs from the other clusters by exhibiting the highest AFP levels, indicative of an aggressive tumor-driven profile. As a result, this cluster analysis clearly reveals the 2 primary causes associated with higher mortality risk in HCC patients, namely, liver dysfunction and tumor progression.

## Discussion

This study aimed to provide a decision-support model for optimizing organ allocation, with a focus on patients with HCC. This is motivated by the challenge that arises from the dual risk of liver failure and tumor burden [12]. Therefore, the management of HCC patients requires a comprehensive approach that simultaneously addresses both oncological and hepatic considerations to optimize clinical outcomes. In this respect, we proposed an advanced EL-based methodology, coupled with SHAP to finely assess the risk of death on the waiting list within 3 months. Our approach allowed the derivation of a probabilistic mortality score, ELM-HCC, which we evaluated in comparison to multiple existing scores.

### Comparison with existing prognostic scores

The results showed that the AFP score, which focuses solely on tumor-related factors, performs the worst, reflecting its inability to capture the competing risk of liver failure. In contrast, scores that incorporate liver dysfunction factors demonstrated better accuracy. Among these, MELD-based scores outperformed Child–Pugh and ALBI scores. Notably, MELD 3.0 provided the best predictive performance, followed by MELD-Na and then MELD. This finding pointed out the pertinence of revising the MELD score by adding serum sodium, albumin, and gender information in MELD 3.0. However, MELD 3.0 was subject to an imbalance between sensitivity and specificity, with specificity considerably exceeding sensitivity, thus limiting its effectiveness in high-risk patient identification. Interestingly, HALT-HCC and the Mehta model outperformed previous scores, likely because they address both key risks for HCC patients. These models integrate MELD-Na, accounting for liver failure risk, while also incorporating AFP and tumor burden to address tumor-related risk. Additionally, the Mehta model includes the Child–Pugh score, providing further insights into factors such as ascites and encephalopathy, which may explain its greater accuracy compared to HALT-HCC.

### Performance of ML models

RF, XGBoost, and LightGBM, when trained on 25 variables, outperformed all previous scores, showing, in addition, a better balance between sensitivity and specificity. This result first demonstrates the ability of such models, fusing several weak learners, in capturing the nonlinear interactions between variables. Secondly, it underscores the interest of considering simultaneously multiple variables, in contrast to previous scoring methods that were restricted to a predefined subset of variables.

### Added value of dynamic variables

When dynamic variables were incorporated (31 variable in total), LightGBM emerged as the top-performing model, achieving an AUROC of 0.831 with a specificity of 73.42% and a sensitivity of 77.42%, demonstrating its strong discriminatory power for identifying high-risk patients. By capturing patients’ health evolution across visits, dynamic variables enable a more comprehensive assessment of both liver dysfunction and tumor progression, thereby enhancing mortality prediction. Our findings supporting the inclusion of additional factors and dynamic variables to enhance LT allocation in HCC patients are in line with recent studies [11,12].

### Feature selection and explainability

Then, the LightGBM, when trained on only the top 8 features selected by SHAP, outperformed the model using all 31 variables. This underscores the high predictive power of the selected features in the decision-making process. Leveraging the strengths of this optimized LightGBM model, we proposed the probabilistic score, ELM-HCC, tailored to predict waitlist mortality risk in HCC patients.

In the medical field, explaining ML-based predictions is crucial yet challenging. Unlike previous studies that only displayed the importance of features [20], our study provided a refined analysis of the identified key features. The selected features were found clinically relevant, representing both the severity of liver disease and the tumor burden of HCC. Indeed, they included MELD components and other factors, such as AFP, largest tumor size, and degree of ascites. Moreover, our results emphasized the importance of monitoring AFP levels over time in mortality risk assessment [47]. Indeed, slightly positive SHAP values when AFP_DIFF is zero suggest that stable AFP levels may still pose a risk, requiring closer monitoring or alternative strategies.

### Risk stratification through SHAP-enhanced clustering

Furthermore, this study showed the effectiveness of SHAP-enhanced clustering for risk stratification. By embedding SHAP values into UMAP and applying *K*-medoids clustering, we retrieved 7 clinically meaningful patient subgroups. This 2-step approach, combining quantitative indices with SHAP-based clinical interpretation, ensured that the chosen number of clusters reflected both statistical validity and clinical relevance. Notably, *K*-medoids produced cluster representatives that correspond to actual patient observations, enhancing interpretability. The analysis of such medoids confirmed the 2 primary pathways associated with high mortality risk in HCC patients: liver dysfunction and tumor progression. Indeed, the highest-risk cluster is dominated by liver dysfunction features, while other groups show tumor-driven risk or mixed clinical profiles. Specifically, high levels of serum creatinine, tumor size, bilirubin, AFP, and moderate ascites were associated with increased risk, whereas lower values corresponded to lower risk. These findings demonstrate how explainable ML can uncover underlying biological pathways and improve patient stratification beyond traditional scores.

### Limitations

Our study suffers from some limitations. One limitation of this study is the absence of external validation. While our internal validation strategy (cross-validation across multiple balanced subsets) supports the robustness of our findings within the UNOS registry, external validation on other cohorts from different healthcare systems (e.g., in Europe or Asia) will be necessary to confirm the generalizability of our model. Also, the UNOS database provides a restricted set of variables defined by clinical practice and prior knowledge; other important prognostic factors are not captured. Incorporating these in future studies may further improve predictive performance. Additionally, missing values were handled using mean imputation. While this simple approach is widely applied in registry-based studies and was appropriate given the low proportion of missing data, we acknowledge that more advanced imputation methods should be investigated in future work. In addition, we focused on predicting 3-month waitlist mortality, which may not capture longer-term risks in HCC. Extending the model to predict longer-term outcomes could offer a more comprehensive prognosis for HCC patients over time. Also, we did not address potential biases related to gender, age, or race; future work should investigate equitable liver allocation across demographic groups. Lastly, although we computed differences between consecutive observations to capture intrapatient changes, this approach does not capture the full evolution of a patient’s health over time. A time-series analysis would provide a more comprehensive view of a patient’s condition, enabling better predictive outcomes.

## Conclusion

Our comprehensive study provided valuable insights into predicting liver transplant waitlist mortality for HCC patients. We proposed a novel score, ELM-HCC, derived from the LightGBM model, that achieves significant improvements over current existing scores for predicting 3-month waitlist mortality risk. Additionally, we identified key risk factors that enhance mortality risk assessment. Moreover, we demonstrated the effectiveness of SHAP-enhanced clustering for a refined mortality risk assessment, providing an in-depth understanding of mortality risk for patients. The meaningful insights raised from SHAP force plots, associated with the representatives of the retrieved profiles, offer clinicians a valuable tool for a more precise risk evaluation, guiding treatment decisions, monitoring patient progression, and improving model explainability. Collectively, these contributions enable more precise and proactive healthcare, enhancing clinical decision-making and supporting better outcomes for LT candidates. Finally, ensuring fairness will be essential for the equitable application of AI-based decision-support tools in transplantation.

## Data Availability

The data reported here were supplied by the United Network for Organ Sharing as the contractor for the Organ Procurement and Transplantation Network (OPTN). The interpretation and reporting of these data are the responsibility of the authors and in no way should be seen as an official policy of or interpretation by the OPTN or the US government.
