# Supplementary material for: Explainable Mortality Prediction for Liver Transplant Candidates with Hepatocellular Carcinoma: A Supervised Clustering Approach
Source: Health Data Sci. 2026 Jan 13;6:0295. doi: 10.34133/hds.0295 (PMC12796095; doi:10.34133/hds.0295)
Supplement: Supplementary 1 — Tables S1 to S3 [file hds.0295.f1.pdf]

Table S1. List of variables.

Table S1: List of the 31 variables used in the study.

#	Feature Name	Feature Description
		**Numerical Features**
1	ALBUMIN	Patient albumin laboratory value at listing
2	BILIRUBIN	Patient total bilirubin laboratory value at listing
3	AFP	Patient alpha-fetoprotein value at listing
4	TUMORNUM	Patient number of tumors
5	INR	Patient INR laboratory value at listing
6	SERUM_CREAT	Patient serum creatinine value at listing
7	SERUM_SODIUM	Patient serum sodium value at listing
8	INIT_AGE	Patient age in years at listing
9	INIT_WGT_KG	Candidate weight in kg at listing
10	BMI_TCR	BMI at listing
11	LARGEST_TUMOR_SIZE	Largest value among the five tumor sizes recorded in the OPTN database, feature engineered
12	ALBUMIN_DIFF	Difference in albumin between two consecutive measurements, feature engineered
13	BILIRUBIN_DIFF	Difference in bilirubin between two consecutive measurements, feature engineered
14	AFP_DIFF	Difference in alpha-fetoprotein between two consecutive measurements, feature engineered
15	INR_DIFF	Difference in INR between two consecutive measurements, feature engineered
16	SERUM_CREAT_DIFF	Difference in creatinine between two consecutive measurements, feature engineered
17	SERUM_SODIUM_DIFF	Difference in serum sodium between two consecutive measurements, feature engineered
		**Categorical Features**
18	DGN_TCR	Primary diagnosis at time of listing
19	ASCITES	Patient ascites at listing
20	DIALYSIS_PRIOR_WEEK	Dialysis in the week prior to serum creatinine test
21	ENCEPH	Patient encephalopathy at listing
22	PREV_TX_ENG	History of previous transplant involving the same organ, feature engineered
23	GENDER	Recipient gender
24	LIFE_SUP_COMB	Combination of LIFE_SUP_TCR, OTH_LIFE_SUP_TCR, VENTILATOR_TCR, feature engineered
25	BACT_PERIT_TCR	Spontaneous bacterial peritonitis at listing
26	DIAB	Diabetes mellitus at listing
27	FUNC_STAT_TCR	Functional status at listing
28	MALIG_TCR	Any known malignancies at listing
29	PREV_AB_SURG_TCR	History of previous upper abdominal surgery at listing
30	PORTAL_VEIN_TCR	History of portal vein thrombosis at listing
31	TIPSS_TCR	Transjugular intrahepatic portacaval shunt at listing

Table S2. Cohort demographics by class.

Table S2: Cohort demographics by class (on waiting list: 31,300 observations, waitlist mortality: 1,053 observations).

**Variables**	**on waiting list**	**waitlist mortality**	**Variables**	**on waiting list**	**waitlist mortality**
Albumin (g/dL)	3.31 ± 0.68	2.95 ± 0.73	Difference in albumin	-0.01 ± 0.35	0.00 ± 0.44
Total bilirubin (mg/dL)	2.84 ± 4.52	7.64 ± 9.79	Difference in bilirubin	0.22 ± 1.74	1.03 ± 3.67
AFP (ng/ml)	80.47 ± 592.00	248.15 ± 924.54	Difference in AFP	-0.40 ± 286.47	-0.17 ± 5.61
INR	1.45 ± 0.65	1.97 ± 1.36	Difference in INR	0.04 ± 0.48	0.19 ± 1.04
Serum creatinine (mg/dL)	1.01 ± 0.53	1.48 ± 1.11	Difference in creatinine	0.02 ± 0.31	0.11 ± 0.73
Serum sodium (mEq/L)	137.12 ± 4.42	134.71 ± 5.89	Difference in sodium	-0.05 ± 2.97	0.11 ± 3.51
Tumor number	1.23 ± 0.54	1.26 ± 0.56	Diabetes mellitus		
Age at registration (years)	60.26 ± 7.29	59.87 ± 7.14	No	20317 (64.91%)	716 (68.00%)
Weight (KG)	86.80 ± 19.03	85.27 ± 18.15	Type I	379 (1.21%)	8 (0.76%)
BMI	29.42 ± 5.48	28.66 ± 5.36	Type II	9905 (31.65%)	295 (28.02%)
Largest tumor size (cm)	1.73 ± 1.43	2.41 ± 1.19	N/A	699 (2.24%)	34 (3.23%)
Ascites			Functional status		
Absent (Ascites_1)	11746 (37.53%)	201 (19.09%)	10%	-	5 (0.47%)
Slight (Ascites_2)	10852 (34.67%)	380 (36.09%)	20%	120 (0.38%)	43 (4.08%)
Moderate (Ascites_3)	2517 (8.04%)	303 (28.77%)	30%	185 (0.59%)	52 (4.94%)
N/A (Ascites_4)	6185 (19.76%)	169 (16.05%)	40%	1287 (4.11%)	62 (5.89%)
Encephalopathy			50%	1989 (6.35%)	99 (9.40%)
Absent	14659 (46.83%)	311 (29.53%)	60%	3741 (11.95%)	126 (11.97%)
Mild (Grade 1–2)	9733 (31.10%)	433 (41.12%)	70%	8229 (26.29%)	288 (27.35%)
Severe (Grade 3–4)	712 (2.27%)	139 (13.20%)	80%	8264 (26.40%)	209 (19.85%)
N/A	6196 (19.80%)	170 (16.14%)	90%	5025 (16.05%)	87 (8.26%)
Transplanted before, Yes	168 (0.54%)	7 (0.66%)	100%	1227 (3.92%)	18 (1.71%)
Gender			No assistance	308 (0.98%)	20 (1.90%)
Male	23212 (74.16%)	815 (77.40%)	Some assistance	55 (0.18%)	7 (0.66%)
Female	8088 (25.84%)	238 (22.60%)	N/A	870 (2.78%)	37 (3.51%)
Life support at listing, Yes	19 (0.06%)	11 (1.04%)	Malignancy history		
Bacterial peritonitis			Yes	17001 (54.32%)	472 (44.82%)
Yes	829 (2.65%)	80 (7.60%)	No	13586 (43.41%)	556 (52.80%)
No	30265 (96.69%)	945 (89.74%)	N/A	713 (2.27%)	25 (2.37%)
N/A	206 (0.66%)	28 (2.66%)	Prior abdo. surgery		
Portal vein thrombosis			Yes	13188 (42.13%)	399 (37.89%)
Yes	2278 (7.28%)	50 (4.75%)	No	17756 (56.73%)	638 (60.59%)
No	28789 (91.98%)	982 (93.26%)	N/A	356 (1.14%)	16 (1.52%)
N/A	233 (0.74%)	21 (1.99%)	TIPSS at listing		
Dialysis prior week, Yes	428 (1.37%)	102 (9.69%)	Yes	1492 (4.77%)	45 (4.27%)
Primary diagnosis			No	29266 (93.50%)	997 (94.68%)
HCC (4400)	12126 (38.74%)	404 (38.37%)	N/A	542 (1.73%)	11 (1.04%)
HCC + Cirrhosis (4401)	19174 (61.26%)	649 (61.63%)			

Table S3. TRIPOD Checklist.

Table S3: TRIPOD Checklist for reporting prediction model development and validation. Adapted from the TRIPOD Statement (`https://www.tripod-statement.org/`).

Section/topic	Item	Checklist item	Location in the manuscript
		**Title and abstract**	
Title	1	Identify the study as developing and/or validating a multivariable prediction model, the target population, and the outcome to be predicted.	Title
Abstract	2	Provide a summary of objectives, study design, setting, participants, sample size, predictors, outcome, statistical analysis, results, and conclusions.	Abstract
		**Introduction**	
Background and objectives	3a	Explain the medical context (diagnostic or prognostic) and rationale, including references to existing models.	Introduction, paragraphs 1–6
	3b	Specify the objectives, including whether this is model development, validation, or both.	Introduction, paragraph 7
		**Methods**	
Source of data	4a	Describe the study design or source of data (registry, cohort), separately for development and validation datasets.	Methods, Study population
Participants	4b	Specify study dates (start/end of accrual, follow-up).	Methods, Study population
	5a	Describe study setting, number and location of centers.	Methods, Study population
	5b	Eligibility criteria.	Methods, Study population
	5c	Treatments received, if relevant.	NA
Outcome	6a	Define the outcome predicted by the model, including how and when assessed.	Methods, Study population
	6b	Report any actions to blind outcome assessment.	NA
Predictors	7a	Define all predictors, how and when measured.	Table S1
	7b	Actions to blind predictor assessment.	NA
Sample size	8	Explain how study size was determined.	Methods, Study population
Missing data	9	Describe handling of missing data, with details of imputation methods.	Methods, Data preprocessing and feature engineering

Continued on next page

Section/topic	Item	Checklist item	Location in the manuscript
Statistical analysis	10a	How predictors were handled in the analyses.	Methods, Data preprocessing and feature engineering/ Statistical analysis
	10b	Type of model, model-building procedures, internal validation.	Methods, Experimental design
	10d	All measures used to assess model performance.	Methods, Evaluation Metrics
Risk groups	11	Provide details on how risk groups were created.	Methods, Supervised Clustering Analysis
**Results**			
Participants	13a	Describe participant flow, including outcome distribution.	Table S2
	13b	Describe participant characteristics (demographics, predictors, missing data).	Table S2
Model development	14a	Number of participants and outcome events in each analysis.	Table S2
	14b	Report unadjusted associations between predictors and outcome, if done.	NA
Model specification	15a	Present full model (coefficients, intercept/baseline survival).	Methods, Hyperparameter Tuning
Model performance	15b	Report performance measures (with CIs).	Results, Risk score performance; Table 2
**Discussion**			
Limitations	16	Discuss study limitations (sample, events, missing data).	Discussion, Limitations
Interpretation	18	Overall interpretation considering objectives, limitations, and other studies.	Discussion, paragraphs 1–8
Implications	19b	Discuss clinical use and implications for future research.	Discussion, paragraphs 1–8
**Other information**			
Supplementary resources	21	Availability of resources (protocol, calculator, data).	Acknowledgments
Funding	22	Source of funding and role of funders.	Acknowledgments
